# Racial and social disparities in the access to automated peritoneal dialysis - results of a national PD cohort

**DOI:** 10.1038/s41598-017-05544-1

**Published:** 2017-07-12

**Authors:** Roberto Pecoits-Filho, Silvia Carreira Ribeiro, Adam Kirk, Helder Sebastião da Silva, Arthur Pille, Ricardo Sprenger Falavinha, Sandro Scolari Filho, Ana Elizabeth Figueiredo, Pasqual Barretti, Thyago Proença de Moraes

**Affiliations:** 1 Pontifícia Universidade Católica do Paraná (PUCPR), Curitiba, Brazil; 2 Wessex Renal and Transplant Service, Queen Alexandra Hospital, Portsmouth, UK; 3 Programa de Pós-Graduação em Medicina e Ciências da Saúde (Nefrologia), Pontifícia Universidade Católica do Rio Grande do Sul (PUCRS), Porto Alegre, Brazil; 4 Universidade Estadual Paulista (UNESP), Botucatu, Brazil

## Abstract

The prevalence of patients on automated peritoneal dialysis (APD) is increasing worldwide and may be guided by clinical characteristics, financial issues and patient option. Whether socioeconomic factors at the patient level may influence the decision for the initial peritoneal dialysis (PD) modality is unknown. This is a prospective cohort study. The primary outcome of interest was the probability to start PD on APD. The inclusion criteria were adult patients incident in PD. Exclusion criteria were missing data for either race or initial PD modality. We used a mixed-model analysis clustering patients according to their PD center and region of the country. We included 3,901 patients of which 1,819 (46.6%) had APD as their first modality. We found a significant disparity for race and educational level with African American patients less likely to start on APD (Odds ratio 0.74 CI95% 0.58–0.94) compared to Whites whilst those with greater educational levels were more likely to start on APD (Odds ratio 3.70, CI95% 2.25–6.09) compared to illiterate patients. Limiting the use of APD in disadvantaged population may be unethical. Demographics and socioeconomic status should not be necessarily part of the decision-making process of PD modality choice.

## Introduction

The choice of the initial renal replacement therapy (RRT) for patients with end-stage renal disease (ESRD) has been in discussion for decades and is considered to be dependent not only on clinical factors but also related to nonclinical variables such as demographic characteristics, socioeconomic status, and race^1–5^. Despite the fact that improved socioeconomic status and implantation of poverty support programs have generated advances in several domains of daily life (schooling, access to healthcare, etc.), racial and social inequality remains common in Brazil. According to recent data published by Mehrotra *et al*. based in large American dialysis cohort, Blacks and Hispanics with ESRD were less likely to start on a home dialysis and even receive kidney transplantation^6^. Interestingly, such differences in the offering of home dialysis were first reported in the US more than a decade ago^5^, although a survey with 271 US nephrologist reported that race was not associated with PD utilization, even after controlling for demographic and clinical characteristics^7^. The impact of race on RRT choice have also been reported in other regions, sometimes with contradictory results compared to the American data, such as the report from ANZDATA that shows the home therapies (particularly PD) use was less likely in White ESRD patients^4^.

Once PD is the therapy of choice, the next decision is which PD modality to start. Indications for PD modality may be related to clinical characteristics, financial issues logistics, and patient’s preference. Patient’s preference has gained significant importance given the lack of evidence to support the superiority of any PD modality on improvement of quality of life^8^. Giving the patient a choice is important particularly because APD gives flexibility for patients and caregivers that have a full-time employment to continue doing their regular activities^9^. In terms of mortality, a recent data based on the BRAZPD cohort shows that APD was associated with a patient survival benefit^10^. However, the majority of large cohort studies and one small clinical trial found no difference in mortality between groups^11–14^.

On this paper, we will focus on APD given our perception that the prevalence of this therapy is growing worldwide compared to CAPD and that this modality could be associated to positive outcomes^15, 16^. To the best of our knowledge no study has ever analyzed if racial and social disparities may impact the utilization of APD. The aim of this study was to investigate the impact of race and social status in the initial modality of PD in a large PD cohort.

## Methods

This is a nationwide prospective study from the BRAZPD II cohort, the characteristics of which have previously been published^16^. The BRAZPD II cohort was launched in December 2004 and followed patients until January 2011. The database comprises data from 122 dialysis centers covering all regions of Brazil. The medical ethical committees of all participating centers approved the study. The number of prevalent patients in each year was correspondent to approximately 65 to 70% of all PD patients in the country. Demographic data collected included age (years), gender, race, cause of end-stage renal disease (ESRD), history and time of pre-dialysis care, family income (minimum wages per month -0 to 2. 3 to 5, 6 to 10, 11 to 20, >20 MW), education level (illiteracy, elementary, secondary and higher), distance from dialysis center (<25, 25 to 50, >50 km), region where patients live, and center experience expressed in patient-year. Clinical data included body mass index (kg/m^2^), blood pressure (BP) (mmHg), presence of edema, and PD modality (Continuous Ambulatory Peritoneal Dialysis [CAPD] or APD).

The definition of patient race was done by the local healthcare team following instructions from a detailed manual, and is presented here according to the division proposed by the World Health Organization. The inclusion criteria for this study were: age > = 18 years old and have started PD during the study period, from now on called incident patients. Exclusion criteria were the presence of any missing data for either race or initial PD modality. Due the small number of Indians in the database (n = 02), they were excluded from the study.

### Statistical analysis

Continuous variables were expressed as mean ± SD or median and interquartile range, while categorical variables (e.g., gender, race, primary renal disease, presence of comorbid conditions, initial therapy, current PD modality, etc.) were expressed as frequencies or percentages. The primary outcome of interest was the probability to start PD with APD. We used two distinct statistical approaches, logistic regression analysis with adjustments for potential confounders with and without interaction terms and a three-level multilevel analysis clustering patients according to their PD center and the center nested in regions to exclude the possibility that the primary indication for APD was guided by center preferences or region particularity. All variables available in the database were tested in a univariate analysis and those with a p value lower than 0.10 were selected to be included in the multivariate analysis, including interaction terms. Results were expressed as coefficients with 95% confidence interval for the probability of start in APD compared to CAPD. Akaike information criteria were used to choose the best-fit survival model. Statistical significance was set at the level of p < 0.05.

### Ethics Statement

This study was performed in accordance with the Declaration of Helsinki, ethical approval was obtained from the Ethical Committee of the Pontificia Universidade Católica do Paraná (approval number 448) after delegation from the National Ethical Committee (process number 25000.187284/2004.01) and all participants provided written informed consent before enrollment.

## Results

### Study population

**Figure 1:**
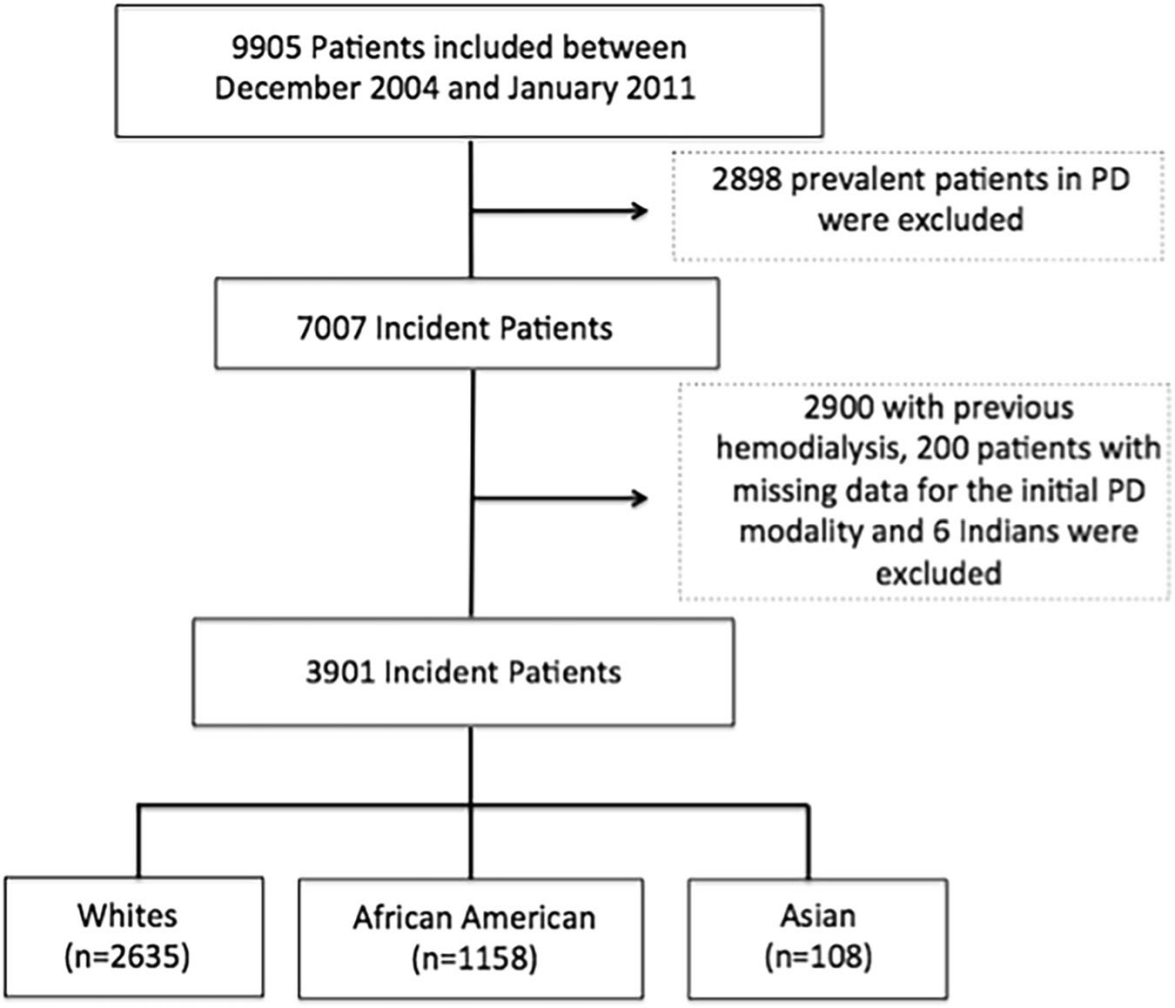
Study population.

This study screened all suitable adult patients starting PD from December 2004 to January 2011. After applying the exclusion criteria, 3901 patients were recruited into the study. Figure 1 depicts patient selection for the study.

### Baseline characteristics

Mean age of the study population was 61.1 ± 16.1; the prevalence of low education levels (<4 years) was 66.2%; family income of less than 2 Brazilian minimal wage per person was 32.6%; diabetes 45.8%; CAD 22.2%; PAD 18.6%; cancer 3.0%; hypertension 73.4%; 51.0% were females; White race was 67.5%, African American 29.7% and Asian 2.8%. The prevalence of African American per regions differed considerably as expected: in the South region was 10.0%, in Southeast 27.4%, Center-west 42.1%, North-east 60.0% and North region 94.0%. Clinical and demographic characteristics according to patient’s race are shown in Table 1. This table illustrates African-American patients presented significant clinical and demographic characteristics compared to White and Asian patients, including a lower duration of formal education and a lower family income. In addition, Table 2 has information about the characteristics of patients starting on CAPD and APD. From the 200 patients excluded for missing initial dialysis modality 72.5% (n = 145) were White, 23% were Black (n = 46) and 4.5% (n = 9) Asian; regarding literacy 68.5% (n = 137) had less than 4 years of formal education.

**Table 1:** Clinical and demographic characteristics according to race.

Variable	Afro-descendant (n = 1158)	White (n = 2635)	Asians (n = 108)
**Primary Renal Disease**			
Hypertension	18.1%	16.2%	7.4%
Diabetes	38.5%	37.6%	63.0%
Glomerulonephritis	7.8%	8.5%	3.7%
Other causes			
Unknown	19.2%	20.4%	13.0%
**Age (years)**	58.8 ± 16.0	62.0 ± 16.2	64.3 ± 14.8
**Body Mass Index** (Kg/m^2^)			
<18.5 Kg/m^2^	7.3%	5.4%	7.4%
18.5 to 25 Kg/m^2^	52.9%	48.8%	50.0%
>25 Kg/m^2^	39.7%	45.8%	42.6%
**Cancer** (yes)	2.7%	3.3%	0.9%
**Centre Experience** (patient-year)	47.0 ± 27.3	44.1 ± 24.7	39.5 ± 25.7
**Coronary Artery Disease** (yes)	18.8%	23.4%	28.7%
**Davies Score**			
0–1	79.4%	72.8%	69.4%
2–3	21.6%	27.2%	30.6%
**Diabetes** (yes)	46.5%	44.5%	70.4%
**Education level**			
≤4 years	70.8%	64.7%	53.7%
**Family Income** (<2 Braz. Min.Wage)	40.9%	29.9%	8.3%
**Gender** (female)	54.6%	49.5%	50.0%
**Hypertension**	76.3%	71.9%	76.8%
**Peripheral Artery Disease** (yes)	18.7%	18.5%	17.6%
**Pre-dialysis Care** (Yes)	59.5%	50.0%	55.6%
**Residual renal function** (yes)	73.1%%	69.5%	76.8%

Differences between groups were not statistically significant only for peripheral artery disease.

**Table 2:** Clinical and demographic characteristics according to initial PD modality.

Variable	CAPD (n = 2082)	APD (n = 1819)
**Primary Renal Disease**		
Hypertension	16.9%	16.2%
Diabetes	35.5%	42.2%
Glomerulonephritis	8.2%	8.1%
Other causes	16.8%	16.8%
Unknown	22.6%	16.7%
**Age (years)** ^**a**^	60.5 ± 15.6	61.8 ± 16.7
**Body Mass Index** (Kg/m^2^)^a^		
<18.5 Kg/m^2^	4.8%	7.5%
18.5 to 25 Kg/m^2^	48.2%	52.2%
>25 Kg/m^2^	47.1%	40.3%
**Cancer** (yes)	3.5%	2.5%
**Centre Experience** (patient-year)^a^		
**Coronary Artery Disease** (yes)^a^	17.7%	27.3%
**Davies Score** ^a^		
0–1	81.4%	74.2%
2–3	18.6%	25.8%
**Diabetes** (yes)^a^	43.0%	45.8%
**Education level** ^**a**^		
1 to 4 years	8.9%	8.6%
4 to 8 years	59.9%	54.0%
>8 years	24.5%	26.7%
**Family Income** (<2 Braz. Min.Wage)	32.3%	32.9%
**Gender** (female)	51.9%	50.0%
**Hypertension**	72.1%	74.8%
**Peripheral Artery Disease** (yes)^a^	82.8%	79.8%
**Pre-dialysis Care** (Yes)	53.5%	52.4%
**Residual renal function** (yes)	69.8%	70.8%

Legend: ^a^p < 0.05.

### Outcomes

There were 1,819 (46.6%) patients starting RRT on APD and we found a significant disparity for race and educational level. Importantly, family income was not associated with the primary outcome. In terms of absolute numbers for race, 1,287 (48.8%) of White patients started on APD, 59 (54.6%) Asians and 473 (40.8%) African Americans; for literacy, 156 (45.7%) of illiterate patients started on APD, 974 (44.0%) with up to 4 years of formal education, 482 (48.8%) with 4 to 12 years and 191 (57.9%) with higher education level.

Covariates that reached statistical significance to be included in all models were year of initiation of PD (hereby called biennium), body mass index (BMI), previous history of cancer, hypertension, peripheral artery disease (PAD) and literacy. Five interaction terms were included in the models with interaction terms: biennium and BMI, coronary artery disease and hypertension, hypertension and diabetes, biennium and literacy, and peripheral artery disease and literacy. Akaike information criterion for the multilevel model (ML) with interaction terms was 5587.427; for the ML model without interaction terms 5613.5; for the logistic regression (LR) model with interaction terms 5156.47; and for the LR without interaction terms 5232.82.

#### Race

**Figure 2:**
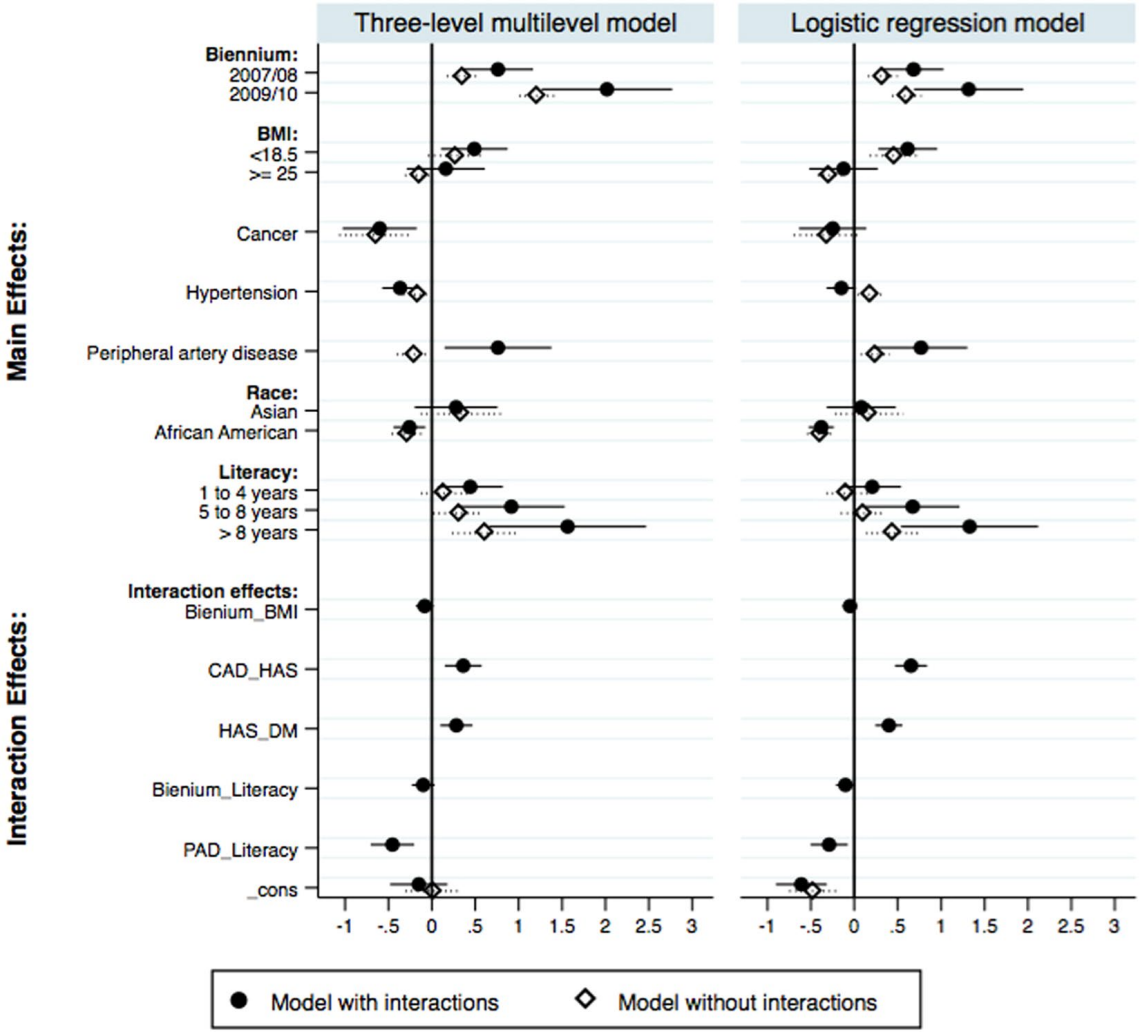
Factors associated with initial PD modality. Values below 0 favour starting on CAPD whilst values above 1 favour starting on APD; the reference group of Biennium was 2005/06; for BMI was 18.5 to <25; for Race was White race; and for Literacy illiterates were the reference group. In the mixed model patients were nested in clinics and clinics nested in regions. Loglikelihood ratio favoured the multilevel model.

African American patients were less likely to start dialysis in APD in all four models compared to White patients: multilevel with interactions (odds ratio 0.77, CI95% 0.64–0.93), multilevel without interactions (odds ratio 0.76, CI95% 0.63–0.91), logistic regression with interaction terms (odds ratio 0.68, CI95% 0.59–0.79) and logistic regression without interactions (0.67, CI95% 0.58–0.77). The same difference was not observed for Asians in any of the models: multilevel with interactions (Odds ratio 1.32, CI95% 0.82–2.13), ML without interactions (Odds ratio 1.41, CI95% 0.88–2.26), LR with interactions (Odds ratio 1.08, CI95% 0.73–1.61), LR without interaction terms (Odds ratio 1.19, CI95% 0.80–1.76). Figure 2 summarizes the coefficients with CI95%of all 4 models.

#### Socioeconomic characteristics

Formal education was positively associated with the odds for APD as the initial PD modality. Compared with illiterate patients, individuals with higher education levels presented a greater odds ratio for start on APD in all models: ML with interaction terms (Odds ratio 4.78, CI95% 1.93–11.80), ML without interactions (Odds ratio1.84, CI95% 1.27–2.67), LR with interaction terms (Odds ratio 3.77, CI95% 1.71–8.30) and LR without interactions (Odds ratio 1.56, CI95% 1.14–2.14). Patients with 5 to 8 years of formal study had a greater odds ratio for start on APD in 3 of 4 models, including: ML with interaction terms (Odds ratio 2.50, CI95% 1.35–4.62), ML without interactions (Odds ratio 1.36, CI95% 1.01–1.82), and LR with interaction terms (Odds ratio 1.96, CI95% 1.14–3.36). For the LR without interactions the result was an odds ratio of 1.11 CI95% 0.86–1.43). Finally, the group of patients with 1 to 4 years of study only presented a greater Odds ratio for start on APD in the multilevel model with interaction terms (Odds ratio 1.56, CI95% 1.07–2.26).

## Discussion

This is the first study to examine and report the influence of racial and social disparities on the initial PD modality in a nationwide cohort. African American patients and those with lower educational level were less likely to start APD, when compared with their white counterparts.

The history of Brazil is closely related to the Atlantic slave trade; slavery officially ended in 1888 but its effects can be felt even nowadays. Moreover, political and economic changes in Brazil also generate socioeconomic disparities. Those disparities deeply affect many aspects of health care, including the prevalence and incidence of RRT^17^. Also, not only indication of the first dialysis therapy was associated with race, but also the probability to receive a kidney transplant was higher for Whites^6^.

First it is important to clarify that APD and CAPD are universally covered by the public health system with no cost for patients. In the small percentage of patients (<10%) that opted for a private health insurance the access for both modalities are also available with no additional cost for patients in addition to the regular monthly taxes. In addition, our health system allows any patient with private health insurance to freely to move to the public health system at any time.

One possible factor influencing sub-modality choice is a selection bias by healthcare workers counseling chronic kidney disease (CKD) V patients. The tree-level multilevel approach was supposed to attenuate such differences if one exists. Moreover, pre-dialysis care, defined as at least 3 months on non-dialysis CKD clinics before initiation of PD, was similar between groups. Further details about how the counseling was done in each clinic are not available.

We then tried to understand whether the differences identified could be explained for any potentially unmeasured confounder including clinical characteristics, such as membrane profile or volume status, and factors such as the universal access to electricity. The indication of PD modality based on membrane profile is a well-established practice^18^. However, this decision is normally taken only after the performance of a peritoneal equilibration test after 30 days of the beginning of PD, which is considered the minimum time required for the peritoneal membrane to adapt to its new environment^19^. In our study no patient had a PET when the decision for the initial PD modality was taken.

Another clinical finding that could have influenced the decision to use APD is related to signs of hypervolemia prior to the initiation of the therapy. Unfortunately a good marker of volume status is not normally available for the greater majority of advanced CKD and dialysis patients in Brazil. Nevertheless, hypertension defined before initiation of PD was a covariate associated with a greater chance of starting on APD. Perhaps some those patients were more likely considered hypervolemic, which in turn would favor short dwells to obtain a higher ultrafiltration volume. In addition, early or late referral to nephrologist was tested but we did not find a significant association with the outcome.

Finally, one could raise the possibility that previous studies, including 2 from the BRAZPD, reported different outcomes for different races, which could have influenced modality selection. However, most of these studies never stratified risk factor for PD modality^20–22^. It is important to reinforce that our previous findings, showing that Asian race presented different chances for peritonitis in APD and that Black race was associated with survival advantage, were published only after the end of data collection and so having no influence in the choice of modality in the present study^23^.

Social characteristics that could have had impact on the choice of APD on developing countries, non-accessibility to electricity may be a concern. In Brazil a national census from 2011 found 2.2% of all homes without electricity. Most of these homes were located in remote areas from the Northeast and North region. This was one of the reasons because adjustment for center-effects and the distance from patients home to the PD center was necessary. Notwithstanding the prevalence of both Black and poor patients is considerably higher than the number of homes without electricity and so, as expected, we found no impact on the results of distance from patient’s home to PD center.

Our data shows a significant increase in the APD utilization as the initial PD modality throughout the study period. Reasons are not clear but may be related to several factors including greater availability of cyclers and a growing advertisement about potential benefits of APD by dialysis companies, mainly pertaining to a better quality of life. Important to say that our previous work showing a better patient survival for individuals on APD compared to CAPD was not available during the study recruitment. Independent of the reasons behind the increase in the use of APD, this seems to be a global trend^15^.

Our study presents some limitations, including the lack of some covariates that could have influenced the choice of initial PD modality such as volume status, marital status, accessibility to electricity, and patient’s opinion. In addition, we don’t have information whether the patient had the treatment paid by the public health system, which represents more than 90% of the whole Brazilian population in dialysis, or by a health private insurance.

In conclusion, despite the improvement in socioeconomic indicators observed in the last decades, including a better access to education and healthcare in Brazil, racial and social inequalities are still present in the country. These inequities appear to impact not only the prevalence of ESRD and the access to RRT, but also impact the utilization of APD. Future studies will be necessary for a better understanding if and how race, educational level and associated socio-economic factors correlates to the potential benefit of APD on patient survival. On a policy level, such approach may help to provide evidence for correction of the factors that could have been caused unequal access to APD. Finally, our intention is not to promote an increase in APD uptake, instead we want to increase awareness about inequalities in the country and limiting the use of APD in disadvantaged population may be harmful to these subgroups. Demographics and socioeconomic status should not be part of the decision making process of PD modality choice.
